# The changing landscape of membrane protein structural biology through developments in electron microscopy

**DOI:** 10.1080/09687688.2016.1221533

**Published:** 2016-09-09

**Authors:** Shaun Rawson, Simon Davies, Jonathan D. Lippiat, Stephen P. Muench

**Affiliations:** ^a^ School of Biomedical Sciences, Faculty of Biological Sciences, University of Leeds, Leeds, UK

**Keywords:** Electron microscopy, membrane protein, protein structure

## Abstract

Membrane proteins are ubiquitous in biology and are key targets for therapeutic development. Despite this, our structural understanding has lagged behind that of their soluble counterparts. This review provides an overview of this important field, focusing in particular on the recent resurgence of electron microscopy (EM) and the increasing role it has to play in the structural studies of membrane proteins, and illustrating this through several case studies. In addition, we examine some of the challenges remaining in structural determination, and what steps are underway to enhance our knowledge of these enigmatic proteins.

## Introduction

The structures and functional mechanisms of membrane proteins are still relatively poorly understood, despite making up approximately 30% of all proteins (Wallin & Heijne, 1998). They play vital roles in cells, from acting as receptors in signalling pathways, allowing passive and active transport of key molecules and ions, to maintaining the proton motive force and synthesis of ATP. It is estimated that 60% of all drugs target membrane bound proteins, including G-protein-coupled receptors (GPCRs), ATP-binding cassette (ABC) transporters, and ion channels, across a wide range of therapeutic areas from antibiotics to cancer (Overington et al., 2006). Indeed, several blockbuster drugs, such as sulphonylureas, benzodiazepines, opioids, and beta blockers all act via membrane protein targets. However, structures of membrane proteins only make up ∼3% of crystal structures in the Protein Data Bank (PDB) and only ∼10% of released structures in the Electron Microscopy Data Bank (EMDB). This reflects on the challenges associated with membrane protein structural biology. The first of which lies in overexpression of the target membrane protein, which can be more difficult than their soluble counterparts with problems associated with cell toxicity, requirement for chaperones, membrane crowding and stability (Seddon et al., 2004). Even with well-expressed membrane proteins there are a number of hurdles to overcome, not least their extraction from the membrane in a stable, non-aggregated state. This has historically relied on detergents, although amphipols and nanodiscs have become increasingly popular, as are new technologies such as styrene maleic acid copolymer lipid particles (SMALPs) and saposin-lipoprotein nanoparticle systems (Bayburt & Sligar, 2010; Breyton et al., 2010; Frauenfeld et al., 2016; Postis et al., 2015; Tate, 2010).

Even with solubilised and purified protein in hand the traditional route of structural determination, X-ray crystallography, is challenging, not least due to the difficulties in forming sufficient crystal contacts required to form well-diffracting protein crystals. This is often caused by the detergents forming large micelles around the membrane protein, which may also exhibit inherent flexibility (Bill et al., 2011). These significant hurdles have been tackled by a number of strategies that include improving protein stability through mutagenesis (Abdul-Hussein et al., 2013), antibody/nanobody binding to increase crystal contacts and/or lock conformational states (Pardon et al., 2014), lipidic cubic phase (LCP) techniques, lipidic bicelles (Ujwal & Bowie, 2011), and the creation of chimeras (Caffrey, 2015; Long et al., 2007; Nishida et al., 2007). However, although these techniques have provided powerful means to obtain crystal structures of membrane proteins, especially GPCRs, they are not a universal solution and many membrane proteins have proven to be intractable to crystallisation despite intense efforts. Therefore alternative approaches are required to tackle those systems that resist crystallisation.

Historically, the determination of membrane protein structures to high resolution by cryo electron microscopy (EM) was only obtainable through electron diffraction of 2D crystals, with the highest resolution single particle EM structure pre-2009 being the RyR1 calcium-release channel at a modest 9.6 Å resolution (Ludtke et al., 2005). This was not just a feature of the EM hardware, but the relatively small size of many membrane proteins of interest and their propensity to aggregate precluded their high resolution structures from being obtained. Thus, historically only very large membrane proteins, such as the rotary ATPase transporters, were routinely studied via EM. Their size and flexible structure meant that crystallographic studies of the whole complex were very demanding, although it is important to note that the full F-ATPase has, after many years, now been solved by crystallographic means (Morales-Rios et al., 2015).

**Figure 1.**
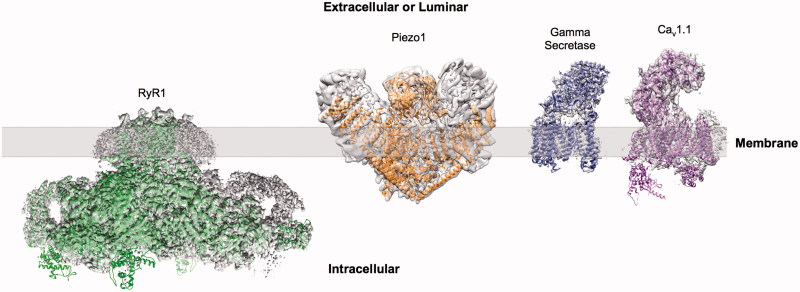
Examples of sub nm membrane protein structures determined by cryo-EM. Examples showing EM reconstructions of RyR1 (EMDB-2807), Piezo1 (EMDB-6343), γ-secretase (EMDB-3061) and Ca_v_1.1 (EMDB-6475) (grey density) with fitted atomic models. This Figure is reproduced in colour in *Molecular Membrane Biology* online.

EM is able to overcome several of the key challenges in membrane structure determination faced by crystallography. While large amounts of protein are required for crystal studies (mg scale), EM can be carried out with a comparatively small amount of protein (μg scale). At the same time, while the presence of detergent can hinder the optimisation of freezing conditions when preparing cryo grids, it does not present a problem to the same extent as in crystal growth, where the resulting micelle can limit crystal contacts (Schulz, 2011). Coupled with recent developments in detectors and microscopes, the use of EM for the study of membrane proteins has been rapidly increasing. Since the widespread adoption of direct detectors several key membrane proteins have had high resolution structures solved by EM including TRPV1, gamma secretase (γ-secretase), RyR1, Ca_v_1.1, NPC1 and piezo (Figure 1) (Bai et al., 2015a; Gao et al., 2016; Ge et al., 2015; Gong et al., 2016; Wu et al., 2015; Yan et al., 2015). Indeed the quality of the EM maps is now sufficient in some cases to permit the identification of a bound ligand or inhibitor as with TRPA1 and γ-secretase (Paulsen et al., 2015; Bai et al., 2015b). But what is behind this change in the landscape of membrane protein structural biology?

## Electron microscopy

In recent years cryo-EM has undergone a step change in its capabilities, moving from being a niche technique used in only a handful of special cases, primarily large (>1 MDa) or extremely symmetrical systems such as viruses, to a widespread high resolution structural method (Bai et al., 2015c; Nogales, 2016). This has been driven through the development of new technologies including better, more stable microscopes, and crucially the introduction of direct electron detectors (DED). An advantage of DED is a much higher Detector Quantum Efficiency (DQE), which is a measure of how efficiently signal is detected at varying resolution ranges. This allows for a higher signal to noise ratio (SNR) and more efficient retention of high resolution information than traditional CCD cameras. The major benefit from this new generation of detectors is the ability to record high frame rate “movies” (Campbell et al., 2012; McMullan et al., 2009, 2014). Practically this means that rather than collecting a single image containing the average of all scattering events over the exposure time, instead we obtain a series of frames, each containing only a small fraction of the total electron dose. In conjunction with the development of these detectors, advances have been made in the algorithms used to process the EM data. Several methods have been developed which can utilise the movie frame recording ability of these detectors to account for mechanical stage drift through computational motion correction (Li et al., 2013; Grant & Grigorieff, 2015a; Rubinstein & Brubaker, 2015). With traditional detectors, which take an average image over the whole of the exposure, this movement would cause blurring of the sample being studied, thus lowering the obtainable resolution or rendering the image useless for structural determination. Moreover, electron beam-induced movements of individual particles in the ice can be tracked and corrected by a number of programs (Grant & Grigorieff, 2015b; Rawson et al., 2016; Rubinstein & Brubaker, 2015; Scheres, 2015). A typical EM exposure of ∼20 e^-^/Å^2^, is required to produce a workable contrast in the microscope. However, radiation damage with doses as low as only ∼3 e^-^/Å^2^ cause significant damage to some amino acids (Grant & Grigorieff, 2015a), which is further discussed below. By removing those frames collected at the end of the exposure or by weighting each frame according to the dose received, one can mitigate to some extent the highly damaging effects of the electron beam on the specimen. What effects have these new technologies had on the membrane protein field?

**Figure 2.**
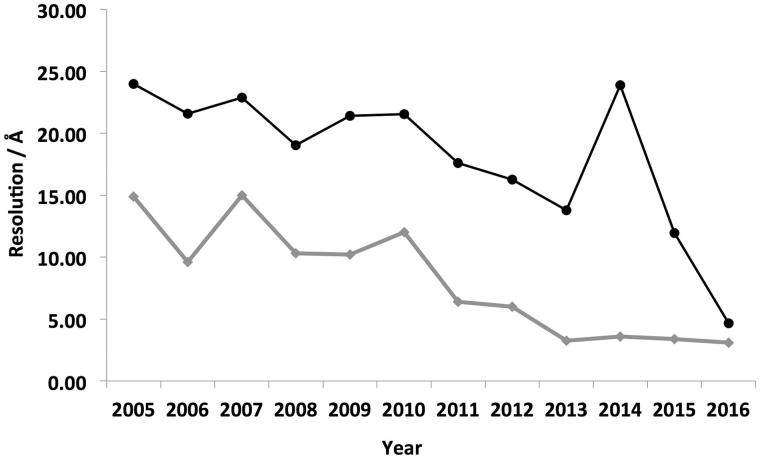
Mean resolution vs year of deposition within the EMDB. Analysis of the average resolution of deposited membrane protein structures within the EMDB in each year is shown in black. Highest resolution reconstruction deposited each year is show in grey. The general trend shows the resolution steadily increasing over time.

In 2006, prior to the development and adoption of these new technologies, there were eight membrane protein structures deposited in the EMDB with an average resolution of ∼22 Å (Figure 2). In contrast, in 2015 the number of deposited structures increased to 52 and the average resolution has improved to 12 Å (Figure 2). It is important to note that not all published membrane proteins have been deposited within the EMDB, but this does provide an approximate representation of general trends. If the current trends were to continue then it is tempting to speculate that EM could become the structural technique of choice for a wide range of membrane proteins in the future whose MW exceeds 100 kDa.

## Membrane protein case studies

A large range of membrane protein structures have now been solved by single particle cryo-EM and the following section aims to provide an overview, but is by no means an exhaustive list.

γ-secretase is an intramembrane protease which acts by cleaving single pass transmembrane proteins at a site within the transmembrane domain. It is implicated in Alzheimer’s disease whereby its cleavage of amyloid precursor protein can give rise to the toxic, abnormally folded, and amyloidogenic Aβ protein. Therefore, it has been the focus of several drug discovery programs (Imbimbo, 2008), but despite the availability of protein there have been no high resolution crystal structures obtained. In 2014 the first high resolution structure of the complex was shown via EM at 4.5 Å resolution, showing details of the transmembrane region and overall architecture (Lu et al., 2014). This was followed up by a subsequent structure, which through using a larger dataset, a higher magnification, and accounting for structural heterogeneity, increased the resolution to 3.4 Å (Bai et al., 2015a). A subsequent paper focusing on the heterogeneity of a single subunit improved the resolution in this area allowing for the direct visualisation of a bound inhibitor to the protein through a combination of signal subtraction and focussed classification (Bai et al., 2015b). Moreover, by identifying different conformational states within the dataset, an insight into the mechanical workings of γ-secretase was obtained. The γ-secretase system provides a good example of how the inherent heterogeneity and flexibility, which can be found within membrane proteins, can result in a loss of high resolution information in these domains, which can be overcome through computational means.

Cryo-electron microscopy has proven to be invaluable in the study of extremely large systems, such as the Piezo1 mechanosensitive cation channel. The Piezo1 channel is a homotrimeric complex with a molecular weight of ∼900 kDa. Piezo1 is expressed in a broad range of tissues such as bladder and lungs, as well as sensory relay cells such as dorsal root ganglial neurons and it is activated by mechanical pressure (Chalfie, 2009; Coste et al., 2010). Mutations in human Piezo have been linked to a number of hereditary disorders, making it a potential target of therapeutics (Albuisson et al., 2013; Bae et al., 2013). A recent structural study of mouse Piezo1 produced a 4.8 Å structure of the channel complex, revealing an architecture that is distinct from all previously-reported ion channel structures. Cryo-EM analysis was used in conjunction with X-ray crystallography to generate the previously unknown peripheral “blade” domains of the protein (Ge et al., 2015). Although the peripheral “blade” regions of the reconstruction are at a significantly lower resolution (∼10–11 Å) than the well-ordered core (∼4.5–6 Å), which prevents accurate building of models in the peripheral domain, the data generated by cryo-EM has shed light on an element of the channel structure that had proven difficult to determine through crystallography.

The structure of the Ca_v_1.1 complex from rabbit skeletal muscle tissue was recently determined via single particle cryo-EM with an average resolution of 4.2 Å (Wu et al., 2015). The L-type calcium channel is a heteropentameric voltage-gated calcium-selective channel that is involved in the contraction of skeletal muscle tissue. The complex comprises five protein components: α_1_, α_2_, β, γ and δ; the α_1_ subunit (in this case Ca_V_1.1) possesses four homologous transmembrane domains, each consisting of six transmembrane helices, which forms the pore-forming domain of the complex. It would otherwise be difficult to study via X-ray crystallography due to its pseudosymmetric nature and heavy glycosylation (Baker, 2010). Ca_v_1.1 is a strong target for drug development, being implicated in hereditary cardiac arrhythmia, epilepsy and hypokalemic periodic paralysis. Inhibiting L-type calcium channels with dihydropyridine drugs has been widely used to treat cardiovascular disorders for several decades.

Another interesting group of membrane proteins that have been extensively studied via EM are the ryanodine receptors (RyR) that act as intracellular Ca^2+^ release channels. As a consequence of RyR’s importance in muscle contraction they have been implicated in several disease states that include cardiac arrhythmias and malignant hypothermia (Betzenhauser & Marks, 2010; Mackrill, 2010) and have therefore emerged as potential therapeutic targets for conditions such as heart failure and myopathies (Andersson & Marks, 2010). Structurally, RyR1 is the largest known ion channel with a total molecular mass greater than 2.2 MDa, and consisting of four protomers each containing over 5000 residues (Yan et al., 2015). Due to its importance and large size several EM studies of RyR1 have been carried out. Prior to the widespread adoption of direct detectors the highest resolution structures obtained were ∼1 nm resolution, showing overall architecture of the complex (Ludtke et al., 2005; Samsó et al., 2009). More recently several EM studies were published in rapid succession with the resolution improving to ∼6 (Efremov et al., 2015), ∼5 (Zalk et al., 2015) and ∼4 Å (Yan et al., 2015), respectively, showing several new features, including identifying the fold of several previously uncharacterized domains, a conformational switch and suggesting potential mechanisms for channel gating.

**Figure 3.**
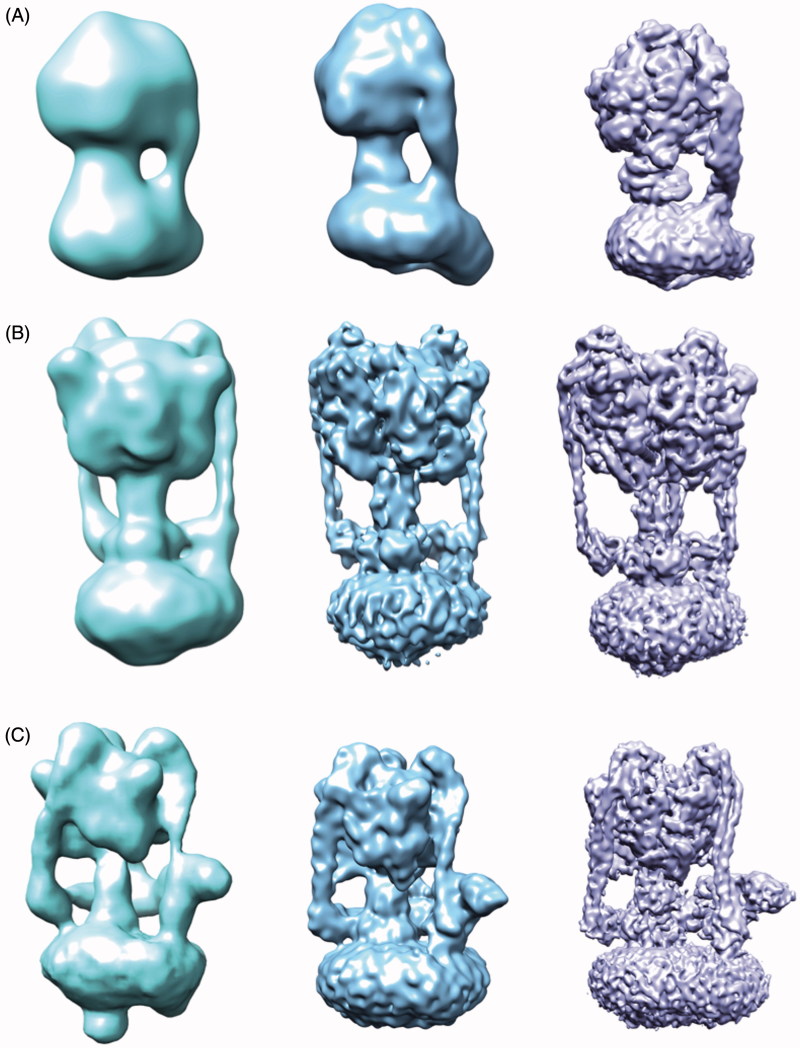
Increasing resolution of the ATPase family structures from EM. (A) F-ATPase structures determined by EM from ∼32 Å to 6.4 Å (EMDB accession codes: 1357,2091,3169). (B) A-ATPase structures determined by EM from ∼16–6.4 Å (EMDB accession codes: 1888,5335,8016). (C) V-ATPase structures determined by EM from ∼17–6.9 Å (EMDB accession codes: 1590,5476,6284). This Figure is reproduced in colour in *Molecular Membrane Biology* online.

The rotary ATPase family comprises the ATP synthase (F-ATPase), vacuolar ATPase (V-ATPase), and archaeal ATPase (A-ATPase), which display differences in structural complexity, as exemplified by the number of stators connecting the proton pump and ATP hydrolysing/synthesising domain (Muench et al., 2011). Whereas the F-ATPase generates ATP from a proton gradient, the V-ATPase hydrolyses ATP in order to drive protons across a membrane and has been extensively studied by a variety of biochemical, biophysical and structural techniques (Muench et al., 2011). In many respects structural studies of the rotary ATPase family have mirrored and followed the history of developments within EM as a whole. As the complex is so large and its mechanism requires significant flexibility, EM has generally been favoured over crystallography to study the intact complex. For example, in single particle cryo-EM studies published between 2011 and 2016, the F-ATPase has improved in resolution from 32–6.4 Å, the V-ATPase from 17–6.9 Å and A-ATPase from 16–6.4 Å, as both preparation of the protein samples as well as the microscope and detectors have improved (Figure 3) (Baker et al., 2012; Benlekbir et al., 2012; Lau & Rubinstein, 2010, 2012; Muench et al., 2009; Rubinstein et al., 2003; Schep et al., 2016; Zhou et al., 2015; Zhao et al., 2015). In 2015 EM studies of the yeast V-ATPase in three states and in a single state from the higher eukaryote *Manduca sexta* were published at sub nm resolution, allowing for new mechanistic insights (Rawson et al., 2015; Zhao et al., 2015). Crucially, the increased resolution permitted the organisation of the previously elusive subunit *a* to be visualised, shedding light onto the mechanism of proton translocation within the complex. A similar feature was also observed in EM structures of F-ATPase dimers from *Polytomella* sp. and the A-ATPase, showing this arrangement may be conserved across the rotary ATPase family (Allegretti et al., 2015; Schep et al., 2016). The ability to resolve the structure of the V-ATPase in defined states for the A-, V- and F-ATPase shows the power of EM for membrane proteins where multiple catalytic states can greatly improve our mechanistic understanding.

Although great strides have been made in the EM field the resolution is still not at the level for many X-ray crystallography studies. What are the current limitations and where do the future challenges lie?

## Current limitations/challenges

While advances in hardware and software within EM have enabled it to become a viable and valuable technique for structural studies of membrane proteins several challenges still remain. There will always be some of the same struggles faced by crystallography in obtaining and purifying sufficient amounts of protein and ensuring protein stability. A further complication is the need to solubilise the protein in detergent with several EM studies demonstrating that the choice of detergent plays a large role in the final resolution obtainable and indeed whether the project is at all viable (Gao et al., 2016; Hauer et al., 2015; Lu et al., 2014). While this is an active area of research, several new methods such as amphipol and SMALP have been developed to mitigate this problem. However, it is likely that there will be no “one size fits all” general solution and the method of solubilisation will have to be optimised for each membrane protein in turn (Dörr et al., 2016; Kleinschmidt & Popot, 2014). One important factor, as shown by the recent paper by Cheng and co-workers is that the phospholipids can play an implicit role in stability, mechanism and inhibitor binding (Gao et al., 2016).

A further contributing factor that limits the achievable resolution is the inherent plasticity in the protein sample. The heterogeneity can be broadly split into two categories; a continuous motion or a series of discreet states. In the case where the system is undergoing a series of discrete steps it can be possible to identify each one of these states as exemplified by the presence of three distinct catalytic states in the V-ATPase (Zhao et al., 2015). In the case of the F-ATPase, these three catalytic states could be further subdivided into seven distinct states which provides insights into the catalytic cycling of these rotary motors (Zhou et al., 2015). However, in addition to catalytic heterogeneity that can result in defined sub-states of a protein or protein complex, there may also be a continuous motion between the different domains. For example, the V-ATPase has been shown to undergo flexing of the ATP hydrolysing domain and proton pumping domain (Song et al., 2013). To account for this flexing, it is possible to focus refinement on a single domain/region which can result in significant improvements in resolution of the area of interest (Rawson et al., 2016). However, it should be noted that due to the nature of the flexibility, fixing one domain relative to the other results in reduced quality in the surrounding regions. While methods are being developed to account for this *in silico*, including complex algorithms analysing continuous trajectories of complex molecular machines (Dashti et al., 2014; Frank & Ourmazd, 2016), it will be more generally necessary to optimise the specimen biochemically, perhaps by trapping in a defined state, or through finding a more stable homologue from another species.

The major factor that prevents EM from attaining truly atomic sub 2 Å structures is radiation damage. During the imaging process the specimen is exposed to 20–100 e^-^/Å^2^ of high energy electrons, and prolonged exposure to the electron beam is equivalent to a nuclear detonation at the specimen scale (Glaeser & Taylor, 1978; Orlova & Saibil, 2011). The damaging effects of these high energy electrons are well established, with previous studies showing the loss of diffraction from 2D and 3D crystals demonstrating that the dose limit for high resolution signal was ∼10 e^-^/Å^2^ (Baker et al., 2010). Subsequent experiments on single particles suspended in vitreous ice showed the ablation of high resolution features even at extremely small electron doses of ∼3 e^-^/Å^2^ for some charged side chains (Grant & Grigorieff, 2015a). This dose is typically achieved within the first few frames of a direct detector movie, but these frames are not generally useful for processing due to initial beam induced particle motion, despite containing the majority of the very high resolution signal. Upon initial exposure to the beam a series of poorly defined events occurs, including contraction/expansion of the ice layer itself, the carbon support film and the metal mesh of the grid, causing large abrupt movements, and rendering these frames blurred and unusable. It is thought that if the information from these early frames could be reliably recovered it would be possible for EM to obtain even higher resolutions from significantly smaller datasets than are currently required (Glaeser, 2016). Work is ongoing developing potential solutions including low level pre-irradiation of the sample or improved sample supports, but as the cause of this apparent movement is poorly understood no solution has yet been found.

A further drawback of EM structural studies is the timescale from sample preparation to obtaining the high resolution structure. While it is possible to obtain useful information extremely quickly via EM, often within days for negative stain projects, high-resolution cryo-EM structures generally take a significant period of time, varying between weeks for simple systems to several months for more challenging samples (Thompson et al., 2016). Despite sample preparation often being more rapid than crystal trials and growth, the process of data acquisition and processing are much slower than their crystallographic counterparts. Many high resolution structures of membrane proteins are obtained from datasets containing anywhere between 100,000 and 1,000,000 particles, representing thousands of micrographs. Manual data collection by hand only allows ∼500 micrographs to be collected in a 24-hour session, but increased use of automated data collection software has increased this to ∼1000 micrographs a day. Nevertheless, this still represents several days of data collection and microscope time in order to obtain a sufficient dataset, often several thousand micrographs in challenging cases, for a high-resolution reconstruction.

Data processing is also a significant challenge for EM, both in time required and the amount of data obtained. With the introduction of direct detectors, the image file sizes have substantially increased, with a single exposure leading to 1–2 GB of frame data. Combined with the number of micrographs needed to obtain high resolution this can lead to single datasets taking up to 7 TB of storage, representing a significant challenge and requiring the implementation of appropriate computational infrastructure (Thompson et al., 2016). Other than simply storage, the data processing requires substantial computational resources (Cianfrocco & Leschziner, 2015), with newer maximum likelihood based data processing programs, for example RELION, needing large numbers of CPUs and large amounts of RAM to run at a reasonable speed, even then taking up to a week for some processing tasks.

Additionally, as cryo-EM is a technique still in its infancy there are fewer well-established protocols and processing pipelines available than with 3D crystallography, indeed even the criterion for what determines resolution in the field has been an area of active discussion and debate. As the “rules” have yet to be fully agreed upon there are still several approaches to map validation between individual groups (Patwardhan et al., 2012). Work is underway to standardise the field and to integrate all of the pieces of popular processing software in a similar fashion to CCP4 in the X-ray community, indeed several processing pipelines are under active development to aid this, including Appion, CCP-EM and Scipion (Lander et al., 2009; la Rosa-Trevín et al., 2016; Wood et al., 2015).

## Future potential

While significant limitations and challenges still exist in using EM for the study of membrane proteins, there is a huge amount of future potential and progress being made in the field. Technology is constantly improving, with iterative improvement in microscope stability and coherence tied to ongoing development in detector technology, highlighted by the addition of “counting mode” (an image acquisition mode that allows for even higher SNR images to be recorded) to all the major detector brands. While computation is currently a bottleneck in comparison to X-ray crystallography, graphics processing unit (GPU) acceleration of these tasks is an area of active research, with accelerated programs already available for several steps of the process including contrast transfer function parameter determination, particle picking and motion correction as well as active development in accelerating the currently slow classification and refinement process utilising the same GPU technology (Zhang, 2016). Combined with the constant increase in general computing power this has already reduced the time for some tasks from days to hours or minutes and this trend is only likely to continue, making the “on the fly” processing so familiar to crystallographers a real possibility for EM in the future.

As well as general ongoing improvements to microscopes, detectors and computational methods there are several other developments under way which will enhance EM for membrane proteins. In particular new advances including phase plate technology (Khoshouei et al., 2016), on grid purification (Yu et al., 2016), and *in situ* studies (Wang & Sigworth, 2009) stand to make EM the premier structural technique for the study of membrane proteins. A significant issue with small proteins in EM (<100 kDa) is the low SNR at the dose and defocus ranges commonly used leading to difficulty in alignment. The need to work at defocus is to obtain phase contrast, however high defocus values lead to the suppression of high resolution information, which can be partially restored computationally. Work on the development of phase plates will allow smaller proteins to be studied in the future by drastically increasing the contrast available and allow imaging to be carried out at extremely low defocus (Danev et al., 2014). While phase plates are still in their infancy already they have aided in determining the structure of human Prx3 (∼250 kDa) to 4.4Å resolution and show much promise for allowing even smaller proteins to be studied (Khoshouei et al., 2016). To date the smallest sub nm single particle structure of a membrane and soluble protein is ∼160k Da and 93 kDa, for the human TAP transporter (Oldham et al., 2016), and Isocitrate dehydrogenase (Merk et al., 2016), respectively.

A key challenge for studying many membrane proteins is the difficulties inherent in their overexpression and purification leading to only small amounts of the protein being available. While EM requires relatively little protein, this is often still too much for many interesting membrane proteins, which are not amenable to overexpression. To overcome this, work is underway to develop on grid purification methods so that only the protein of interest is captured on the EM grid and then can be directly studied, removing the need for prior purification. Initial studies have focused on applying Ni^2+^ doped lipid monolayers to the grid surface to extract polyhistidine-tagged protein directly or alternatively immobilising antibodies to the grid surface to tether the protein of interest to the grid (Benjamin et al., 2016). Indeed this approach has already been successful in the high resolution study of viruses (Yu et al., 2016), and it is hoped that this could be applied to many challenging classes of membrane proteins such as poorly expressed channels including neuroreceptors, for example P2X7 (Hughes et al., 2007).

Despite all the recent advances in EM leading to improved resolutions, the structures of membrane proteins that are obtained still suffer from a crucial drawback. As with crystallography the protein structures are invariably obtained following purification and stabilisation within a detergent. While great progress has been made in producing and finding milder and more relevant detergents they are still significantly different from the native membrane environment. Indeed, the function of proteins is known to undergo large changes upon purification within a detergent (Postis et al., 2015) casting some doubt on the physiological relevance of some of the membrane protein structures currently available from both EM and crystal studies. Several groups have been developing methodology to combat this lack of native lipids, of particular interest are the efforts to introduce a more native environment through the use of SMALPs and the progress being made in imaging proteins *in situ*, within a vesicle (Dörr et al., 2015; Frauenfeld et al., 2016; Lee et al., 2016; Wang & Sigworth, 2009, 2010). These *in situ* studies would not only allow protein activity to be measured in the imaged sample, but also potentially allow different conformations of the protein to be observed by inducing a membrane potential or a chemical gradient through the addition of additives to the sample. A further consideration is the significantly increasing power of the use of electron tomography, which allows objects on a larger length scale, for example cells, to be visualised. Although out of the scope of this review this technique offers great promise for the direct visualisation of membrane proteins within their native cellular environment (Dodonova et al., 2015; Sharp et al., 2016), although at present only moderate resolution sub nm structures are available through sub tomogram averaging for non-symmetric structures. We would refer the readers to the following reviews, which provide an overview for this growing and exciting area of electron microscopy (Briggs, 2013; Lučić et al., 2013).

While still in its infancy, EM is becoming a key technique for the determination of membrane protein structure. Recent advances in the field have led to near atomic structures becoming routine, and new developments will only increase the resolution obtainable and widen the range of samples that can be studied in this way. While crystallography has proved to be a powerful tool over time for the study of membrane proteins, EM will allow the study of these vastly important proteins from drastically smaller amounts of protein and allow the study of proteins within the native environment (membrane and protein) and in a variety of conformational states, even for targets considered intractable for crystal studies.
